# Supplementary material for: Characterisation of microsatellite and SNP markers from Miseq and genotyping-by-sequencing data among parapatric Urophora cardui (Tephritidae) populations
Source: PeerJ. 2017 Aug 14;5:e3582. doi: 10.7717/peerj.3582 (PMC5560233; doi:10.7717/peerj.3582)
Supplement: Appendix S1 [file peerj-05-3582-s001.docx]

**Appendix S1.**

# Johannesen J, Fabritzek AG, Ebner B, Bikar S-E. Characterisation of microsatellite and SNP markers from Miseq and genotyping-by-sequencing data among parapatric *Urophora cardui* (Tephritidae) populations

**Barcode adapters,** restriction enzyme EcoR1: 5- G’AATT’C -3

| Name | Barcode | BC_N_ | Sequence **5‘ –> 3‘** |
| --- | --- | --- | --- |
| **BC-18-EcoR1** | **taggaa** | 6 | **AATTttccta**AGATCGGAAGAGCGTCGTGTAGGGAAAGAGTGT |
|  |  |  |  |
| **BC-20-EcoR1** | **cttgctt** | 7 | **AATTaagcaag**AGATCGGAAGAGCGTCGTGTAGGGAAAGAGTGT |
|  |  |  |  |
| **BC-24-EcoR1** | **tagcatgc** | 8 | **AATTgcatgcta**AGATCGGAAGAGCGTCGTGTAGGGAAAGAGTGT |
|  |  |  |  |
| **BC-25-EcoR1** | **tgcaagga** | 8 | **AATTtccttgca**AGATCGGAAGAGCGTCGTGTAGGGAAAGAGTGT |
|  |  |  |  |
| **BC-26-EcoR1** | **aaccgag** | 7 | **AATTctcggtt**AGATCGGAAGAGCGTCGTGTAGGGAAAGAGTGT |
|  |  |  |  |
| **BC-JJ1-EcoR1** | **ttgcga** | 6 | **AATTtcgcaa**AGATCGGAAGAGCGTCGTGTAGGGAAAGAGTGT |
|  |  |  |  |

**Common adapters 5‘ –> 3‘**

| Common top  **Com-Top- EcoR1** | **AATT**AGATCGGAAGAGCACACGTCTGAACTCCAGTCA |
| --- | --- |
| Common bottom  **Com-Bottom** | TGACTGGAGTTCAGACGTGTGCTCTTCCGATCT |

**PCR primers with Index ID barcodes** (underlined = Barcode (1) & common bottom (2) sequences)

| Primer | Sequence 5’ –> 3’ |
| --- | --- |
| PCR Primer 1 **GBS P1 univ** | AATGATACGGCGACCACCGAGATCTACACTCTTTCCCTACACGACGCTCTTCCGATCT |
|  |  |
| PCR Primer 2 |  |
| **TS08** (acttga) | CAAGCAGAAGACGGCATACGAGAT**tcaagt**GTGACTGGAGTTCAGACGTGTGCTCTTCCGATC |
| **TS13** (agtcaa) | CAAGCAGAAGACGGCATACGAGAT**ttgact**GTGACTGGAGTTCAGACGTGTGCTCTTCCGATC |
| **TS14** (agttcc) | CAAGCAGAAGACGGCATACGAGAT**ggaact**GTGACTGGAGTTCAGACGTGTGCTCTTCCGATC |
| **TS15** (atgtca) | CAAGCAGAAGACGGCATACGAGAT**tgacat**GTGACTGGAGTTCAGACGTGTGCTCTTCCGATC |
| **TS16** (ccgtcc) | CAAGCAGAAGACGGCATACGAGAT**ggacgg**GTGACTGGAGTTCAGACGTGTGCTCTTCCGATC |
