# Supplementary material for: Characterisation of microsatellite and SNP markers from Miseq and genotyping-by-sequencing data among parapatric Urophora cardui (Tephritidae) populations
Source: PeerJ. 2017 Aug 14;5:e3582. doi: 10.7717/peerj.3582 (PMC5560233; doi:10.7717/peerj.3582)
Supplement: Appendix S2 — Extraction method: (1) DNA extraction from ultrasound-lysed tissue without application of RNAase, (2) DNA extraction from whole-cut insect with application of RNAase. DNA concentration (ng/µl) was measured with Qubit. DNA purity (260/280) was measured with NanoDrop. [file peerj-05-3582-s002.docx]

**Appendix S2**

# Johannesen J, Fabritzek AG, Ebner B, Bikar S-E. Characterisation of microsatellite and SNP markers from Miseq and genotyping-by-sequencing data among parapatric *Urophora cardui* (Tephritidae) populations

DNA extraction method and DNA quality. Extraction method: 1) DNA extraction from ultrasound-lysed tissue without application of RNAase, 2) DNA extraction from whole-cut insect with application of RNAase. DNA concentration (ng/μl) was measured with Qubit. DNA purity (260/280) was measured with NanoDrop.

| Taxon | Extraction method | ng/μl DNA | 260/280 |
| --- | --- | --- | --- |
| Vind_U616 | 2 | 29 | 1.81 |
| Vild_U43 | 1 | 101 | 2.09 |
| Vild_U44 | 1 | 90 | 2.09 |
| Vild_U45 | 1 | 77 | 2.05 |
| Vild_U614 | 2 | 82 | 1.79 |
| Vild_U615 | 2 | 31 | 1.88 |
| Froe_U605 | 2 | 66 | 1.86 |
| Froe_U606 | 2 | 23 | 1.91 |
| Froe_U607 | 2 | 35 | 1.95 |
| Froe_U608 | 2 | 60 | 1.85 |
| Froe_U609 | 2 | 31 | 1.89 |
| Froe_U610 | 2 | 50 | 1.84 |
| NMS_U28 | 1 | 39 | 2.05 |
| NMS_U29 | 1 | 49 | 2.08 |
| NMS_U31 | 1 | 95 | 2.11 |
| NMS_U32 | 1 | 59 | 1.98 |
| NMS_U33 | 1 | 108 | 2.09 |
| NMS_U35 | 1 | 48 | 2.05 |
