# Supplementary material for: Characterisation of microsatellite and SNP markers from Miseq and genotyping-by-sequencing data among parapatric Urophora cardui (Tephritidae) populations
Source: PeerJ. 2017 Aug 14;5:e3582. doi: 10.7717/peerj.3582 (PMC5560233; doi:10.7717/peerj.3582)
Supplement: Appendix S3 — Sum of variable sites = 3643, total number of loci containing parsimony sites = 2476, number of unlinked SNPs = 1492. The loci were based on the restriction enzyme EcoR1. dpt.me = mean depth of clusters, dpt.sd = standard deviation of cluster depth, d > 7.tot = number of clusters with depth greater than 7, d > 7.me = mean depth of clusters with depth greater than 7, d > 7.sd = standard deviation of cluster depth for clusters with depth greater than 7, nloci = number of loci, f1loci = number of loci with >N depth coverage, f2loci = number of loci with >N depth and passed paralog, nsites = number of sites across f loci, npoly = number of polymorphic sites in nsites, poly = frequency of polymorphic sites, He = expected heterozygosity. [file peerj-05-3582-s003.docx]

**Appendix S3**

# Johannesen J, Fabritzek AG, Ebner B, Bikar S-E. Johannesen J, Fabritzek A, Ebner B, Bikar S-E. Characterisation of microsatellite and SNP markers from Miseq and genotyping-by-sequencing data among parapatric *Urophora cardui* (Tephritidae) populations

Genotyping-by sequencing summary of coverage and loci identified by pyRAD 3.0.3 for parameters mincov = 14, maxSH = 6. Sum of variable sites = 3643, total number of loci containing parsimony sites = 2476, number of unlinked SNPs = 1492. The loci were recovered and based on the restriction enzyme EcoR1.

|  |  |  |  |  |  |  |  |  |  |  |  |  |  |  |  |  |  |
| --- | --- | --- | --- | --- | --- | --- | --- | --- | --- | --- | --- | --- | --- | --- | --- | --- | --- |
| Taxa | Nreads | Passed | dpt.me | dpt.sd | d>7.tot | d>7.me | d>7.sd | nloci | f1loci | f2loci | nsites | npoly | poly | He |  | Loci_final |  |
| 02_Vind_U616 | 1104856 | 1095591 | 28.772 | 235.858 | 5077 | 45.159 | 300.083 | 8282 | 5049 | 4773 | 465719 | 1687 | 0.0036224 | 0.00834336 |  | 2507 |  |
| 03_Vild_U43 | 1385236 | 1364767 | 35.632 | 267.265 | 5824 | 52.882 | 328.058 | 8846 | 5794 | 5436 | 546669 | 1879 | 0.0034369 | 0.00920857 |  | 2578 |  |
| 03_Vild_U44 | 1298071 | 1277271 | 32.365 | 249.944 | 5473 | 51.062 | 317.209 | 8895 | 5441 | 5102 | 506414 | 1898 | 0.0037479 | 0.00925384 |  | 2540 |  |
| 03_Vild_U45 | 1135722 | 1118442 | 28.351 | 218.62 | 5286 | 45.078 | 279.323 | 8708 | 5255 | 4926 | 480566 | 1722 | 0.0035833 | 0.00911052 |  | 2533 |  |
| 03_Vild_U614 | 645164 | 639276 | 18.560 | 142.527 | 3934 | 31.763 | 194.15 | 7373 | 3908 | 3674 | 348272 | 1406 | 0.0040371 | 0.0095684 |  | 2153 |  |
| 03_Vild_U615 | 1340948 | 1327897 | 32.978 | 276.373 | 5441 | 53.204 | 354.809 | 9041 | 5415 | 5090 | 496840 | 1806 | 0.003635 | 0.00873002 |  | 2549 |  |
| 16_Froe_U605 | 1350628 | 1338695 | 33.358 | 261.528 | 5533 | 50.468 | 324.806 | 8601 | 5499 | 5200 | 518923 | 1860 | 0.0035844 | 0.00861923 |  | 2565 |  |
| 16_Froe_U606 | 1018495 | 1008436 | 23.755 | 212.829 | 4709 | 40.716 | 285.333 | 8531 | 4685 | 4387 | 416564 | 1652 | 0.0039658 | 0.00926384 |  | 2382 |  |
| 16_Froe_U607 | 701709 | 695141 | 18.686 | 151.549 | 3806 | 34.081 | 213.246 | 7615 | 3783 | 3514 | 326344 | 1285 | 0.0039376 | 0.01010517 |  | 2064 |  |
| 16_Froe_U608 | 1503478 | 1488980 | 38.057 | 309.842 | 5549 | 58.099 | 385.917 | 8673 | 5517 | 5212 | 513802 | 1928 | 0.0037518 | 0.00884911 |  | 2559 |  |
| 16_Froe_U609 | 1149050 | 1137708 | 28.260 | 242.807 | 5111 | 45.445 | 312.85 | 8549 | 5083 | 4794 | 463187 | 1758 | 0.0037954 | 0.00877184 |  | 2523 |  |
| 16_Froe_U610 | 1060716 | 1050945 | 28.738 | 212.904 | 5122 | 43.322 | 265.113 | 8009 | 5089 | 4814 | 478292 | 1750 | 0.0036589 | 0.00860901 |  | 2514 |  |
| 19_NMS_U28 | 2640743 | 2601358 | 56.611 | 502.310 | 6340 | 88.131 | 628.759 | 10002 | 6310 | 5858 | 587663 | 2193 | 0.0037317 | 0.01047367 |  | 2589 |  |
| 19_NMS_U29 | 1367765 | 1352545 | 35.884 | 319.811 | 5000 | 59.702 | 417.434 | 8585 | 4975 | 4662 | 431032 | 1595 | 0.0037004 | 0.00976391 |  | 2463 |  |
| 19_NMS_U31 | 2148088 | 2113420 | 49.877 | 374.497 | 6242 | 76.149 | 464.15 | 9675 | 6210 | 5767 | 570865 | 2193 | 0.0038412 | 0.01033802 |  | 2589 |  |
| 19_NMS_U32 | 2164352 | 2136598 | 49.999 | 421.474 | 5972 | 76.748 | 524.542 | 9317 | 5940 | 5542 | 542689 | 1983 | 0.003654 | 0.00977491 |  | 2585 |  |
| 19_NMS_U33 | 799359 | 789862 | 23.106 | 192.364 | 4244 | 39.593 | 258.851 | 7754 | 4214 | 3947 | 362981 | 1256 | 0.0034602 | 0.00873098 |  | 2191 |  |
| 19_NMS_U35 | 1117890 | 1101393 | 27.727 | 223.879 | 5299 | 43.315 | 284.077 | 8599 | 5269 | 4968 | 497428 | 1859 | 0.0037372 | 0.00906444 |  | 2533 |  |

dpt.me = mean depth of clusters, dpt.sd = standard deviation of cluster depth, d>7.tot = number of clusters with depth greater than 7, d>7.me = mean depth of clusters with depth greater than 7, d>7.sd = standard deviation of cluster depth for clusters with depth greater than 7, nloci = number of loci, f1loci = number of loci with >N depth coverage, f2loci = number of loci with >N depth and passed paralog, nsites = number of sites across f loci, npoly = number of polymorphic sites in nsites, poly = frequency of polymorphic sites, He = expected heterozygosity.
